# Microfluidics in Gas Sensing and Artificial Olfaction

**DOI:** 10.3390/s20205742

**Published:** 2020-10-09

**Authors:** Guilherme Rebordão, Susana I. C. J. Palma, Ana C. A. Roque

**Affiliations:** UCIBIO, Chemistry Department, School of Science and Technology, NOVA University of Lisbon, Campus Caparica, 2829-516 Caparica, Portugal; g.rebordao@campus.fct.unl.pt

**Keywords:** microfluidics, gas sensing, artificial olfaction, volatile organic compounds

## Abstract

Rapid, real-time, and non-invasive identification of volatile organic compounds (VOCs) and gases is an increasingly relevant field, with applications in areas such as healthcare, agriculture, or industry. Ideal characteristics of VOC and gas sensing devices used for artificial olfaction include portability and affordability, low power consumption, fast response, high selectivity, and sensitivity. Microfluidics meets all these requirements and allows for in situ operation and small sample amounts, providing many advantages compared to conventional methods using sophisticated apparatus such as gas chromatography and mass spectrometry. This review covers the work accomplished so far regarding microfluidic devices for gas sensing and artificial olfaction. Systems utilizing electrical and optical transduction, as well as several system designs engineered throughout the years are summarized, and future perspectives in the field are discussed.

## 1. Introduction

Volatile organic compounds (VOCs) can be defined as a family of carbon-containing chemicals that exhibit high vapor pressure at ambient temperature [1]. These organic gases are emitted from a variety of sources and some of them have biological, chemical or physical significance in practical settings, which makes gas sensing, in particular VOC sensing, a growing and highly valued field. For example, VOCs are predominant in urbanized areas as they are released from industrial processes, transportation activities, residences or natural resources [1]. VOCs are the primary source of indoor environmental pollutants [2]. Therefore, monitoring the concentration levels of these vapors is imperative to prevent them from exceeding safe levels, as long-term exposure to certain VOCs has been proven to raise the risk of serious diseases such as cancer and respiratory illnesses [3]. On the other hand, VOCs can also be utilized as biomarkers for the diagnosis and monitoring of different diseases [4,5]. For example, acetone and ethanol are very often found in the breath of diabetics, pentane is abundant in the breath of schizophrenia patients, and high aldehyde concentrations are present in the breath of patients with lung cancer [6]. Production of specific sets of VOCs is also associated with food spoilage, and thus VOC monitoring can be a means for food quality control [7,8,9,10,11]. Hence, developing tools for detecting VOCs, in real-time, in situ, and in a fast and non-invasive manner is of enormous relevance in areas such as environmental monitoring, disease control, security and defense, or in quality control.

Gas chromatography coupled with mass spectrometry (GC-MS) has been the most utilized technique to detect and identify VOCs. Conventional GC, despite the high sensitivity that it provides, possesses several critical drawbacks such as the size and high cost of the equipment, the need for being operated by experts, and the difficulty for in situ detection [12,13,14,15,16,17,18,19]. An ideal VOC-sensing tool should be more user-friendly and circumvent these issues, providing portability, in situ and fast analysis, robustness, and affordability. Artificial olfaction systems mimic the biological sense of smell and usually consist of electronic nose devices (e-noses), which are viable alternatives to the conventional laboratorial gas analysis equipment due to the simplicity of use and lower cost. E-noses are composed by an array of cross-sensitive gas sensors associated with signal processing and pattern recognition algorithms. Typically, there is a sample delivery system (such as pumping mechanism) that takes the gas sample to a hermetic chamber where the sensors are located. There, the interaction of the sample’s VOC molecules with the sensing materials generates signals that are transduced and processed by a computing unit. In the last decade, much work has been devoted to the development of optimized gas-sensors [5,20,21,22], signal processing [23], and device architectures [24,25], always targeting the ideal of a compact, miniaturized e-nose with accurate and selective performance. In particular, important effort has been put on the optimization of the sample preparation and delivery system. Namely, on the incorporation of GC-inspired strategies to pre-concentrate [18,26,27,28,29,30] or separate the components of complex gas mixtures before reaching the sensors [15], to increase system selectivity. For example, different authors have developed an artificial olfactory mucosa which integrates polymer-coated layers, in order to achieve gas-chromatographic separation [31,32,33].

Microfluidics refers to the study of fluids at the submillimeter scale, making use of channels with dimensions of tens to hundreds of micrometers, geometrically confined in small-scale chips. This emerging technology has numerous advantages such as the ability to utilize small quantities of reagents and samples, precisely control experimental conditions, and perform multiplexed processes in an automated and high-throughput manner, resulting in miniaturized devices with high resolution and sensitivity of analysis, low fabrication and operation costs, short analysis time, and able of in situ operation [34,35]. Due to its affordability, transparency, and non-toxicity, polydimethylsiloxane (PDMS) is usually the material of choice to fabricate microfluidic chip devices, with the help of different microfabrication techniques such as photolithography, soft-lithography, and micro-milling [36]. These allow for the design of customized microchannel geometries and for the integration of different stages of sample manipulation in compact devices. Thus, microfluidics technology is an excellent candidate for developing VOC sensing devices based on artificial olfaction. In the context of gas analysis, microfluidics has been used as a tool for sample manipulation prior to analysis with conventional analytical techniques like GC-MS [37], but also integrated with gas sensors to detect VOC analytes released from liquid [14,38] or in gaseous samples [18].

This review aims to cover the work accomplished so far regarding VOC and gas sensing using microfluidic technology and gas sensors. In a gas sensor, the interaction between VOC molecules and the sensing material, be it a chemical reaction or a physical event, is transduced into a measurable signal (e.g., electrical) proportional to the degree of interaction. In this review we focus on miniaturized device designs that employ sensors with electrical or optical transduction. Several efforts to increase the sensitivity and selectivity of the devices are discussed as well. The present review will hopefully provide a starting point for future work regarding VOC and gas sensing using microfluidics.

## 2. Microfluidic Gas Sensing Devices Based on Electrical Transduction

The response of gas sensors that use electrical transduction can be in the form of a variation of voltage, current, conductance, capacitance, resistance or conductivity. The majority of VOC sensors that use electrical transduction are based on metal oxides semiconductors (MOS). The gas-sensing mechanism of MOS gas sensors is based on the chemical reactions that occur on the sensing layer upon exposure to the analytes at elevated temperatures (around 300 °C). This interaction leads to a change in the conductivity of the sensing materials [39], depending on the type of material. When n-type MOS (such as TiO_2_, ZnO, SnO_2_, and WO_3_-based sensors) interact with a reducing gas, there is s an increase in conductivity, whereas if the interaction is with a reducing gas, there is a decrease in conductivity. On the other hand, p-type MOS (such as NiO, Mn_3_O_4_, and Cr_2_O_3_-based sensors) suffer an increase in conductivity when exposed to oxidizing gases, and decrease when exposed to a reducing gas [39]. Due to the advantages of MOS sensors, such as low cost and short response time, they are a popular choice in the field of artificial olfaction, including in microfluidics-based gas-sensing devices. A significant part of reported works consists of GC-inspired architectures, where MOS sensors are coupled to miniaturized components of conventional GC. This section is, thus, divided in GC-inspired approaches and other approaches.

### 2.1. Coupling of Micro-GC (μGC) and MOS Sensors

GC is a bulky and expensive tool despite being a powerful and reproducible method to identify VOCs present in gas samples. MOS gas sensors can be coupled to GC separation columns and replace the usually complex detection methods associated with GC while still taking advantage of VOC separation step [40]. However, this approach alone is not sufficient to reduce the equipment bulkiness. Throughout the past 40 years, the efforts for developing field portable, compact, and low power GC instruments has been significant. Towards that aim, a number of GC systems including miniaturized micro-fabricated components (pre-concentrators, separation columns, micro-heaters) are reported [41], including very compact lab-on-the-chip platforms comprising the sampling system, separation column and detectors like photo-ionization detectors (PID) and thermal coupling detectors (TCD) [42,43]. These are known as micro-GC (µGC) systems. Following this trend, the coupling of MOS sensors with miniaturized components of conventional GC has become a very common approach in the field of microfluidics in artificial olfaction. μGC has a similar working principle to that of conventional GC. Essentially, as different analytes go through a separation column (which here is addressed as μGC column) they endure different processes, and thus exit the column at different times [41]. Different approaches found throughout the literature that couple commercial MOS sensors and µGC columns are addressed and listed in Table 1. Different parameters are presented, such as the coating of the µGC column, the sensing material, the tested analytes and the limits of detection (LOD) achieved with each system.

**Figure 1:**
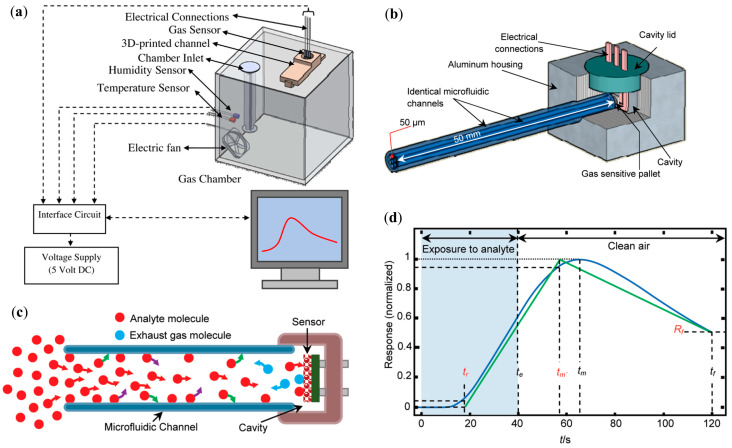
Structures composing the device and line diagram. (**a**) Schematic of the experimental setup. (**b**) Channel-sensor coupling. (**c**) Analyte diffusion along one channel. The red, green, violet, and blue arrows illustrate the diffuse-in, physisorption, desorption, and diffuse-out processes, respectively. (**d**) An experimental normalized response profile and its “line diagram”. The line diagram is specified by determining tr and tm′, moments where the response profile reaches 5% and 95% of its maximum level, respectively, and Rf, the dimensionless magnitude of the profile at tf. (**a**) Reproduced with permission. [17] Copyright 2017, Elsevier. (**b**–**d**) Reproduced with permission from [15] Copyright 2010, American Chemical Society.

Like mentioned before, a common approach for microfluidic VOC detection is the coupling of a µGC column and a generic MOS gas sensor. A chemiresistive sensor based on tin oxide (SnO_2_) [14,15,16,17,45] is an example of such sensor. The typical structure of a chemiresistive metal oxide gas sensor comprises of a ceramic substrate with a thick film metal oxide sensing pallet on one side, and a thermo-resistor micro-heater on the other [17]. An example of the experimental setup is presented in Figure 1a, and the column/channel-sensor coupling can be seen in Figure 1b. The working principle of this device is fairly straightforward: a liquid sample is introduced into a sample chamber, where it evaporates after being placed on a borosilicate glass plate (due to a mini-heater located under the glass plate), filling the gas chamber; analyte molecules exit the chamber and diffuse throughout the length of the channel; once the analyte molecules reach the pallet of the sensor, a response is generated. For the response profile to reset, the channel end that is attached to the analyte-filled chamber is connected to a clean air chamber, and by doing this, the molecules diffuse out of the channel. When analyte molecules first enter the channel, a competition takes place between the phenomena of diffusion (along the channel), adsorption, and desorption (to and from the channel walls) [15,17]. Since each analyte has its specific molecular diffusivity in air, adsorption propensity, and rate of desorption, a different temporal response profile is generated for a different analyte. A typical response profile is shown in Figure 1d. A simplified graphical representation of the response, the “line diagram”, based on three key features of the response profile (tr, tm, Rf in Figure 1) is then used as source of information for sample identification.

A set of analytes were successfully identified at concentrations ranging from 900 to 1100 ppm, such as hydrogen, carbon monoxide, argon, oxygen, methanol, ethanol, isopropanol, 1-propanol, *tert*-butanol, 2-butanol, *iso*-butanol, 1-butanol, methane, *n*-butane, *n*-pentane, acetone, butanone, 2-pentanone, methyl isobutyl ketone, chloroform, toluene, benzene, carbon tetrachloride, and ammonia. Analyte recognition was achieved through a common method, by determining the 3-D “feature vector” (θ(tr, tm, Rf)) of each analyte, representing it in the “feature space”, and comparing it to θs of gases of interest [15]. Essentially, individual VOC distinction was achieved based on the temporal characteristics of the responses. Moreover, the authors reported the ability of the device to successfully estimate the composition of two- and three-component mixtures, by using the same method. Experiments to access the operating range of concentrations were performed with methanol samples, but encountered technical problems below ∼200 ppm, due to low signal levels and above 500 ppm due to loss of linearity of the response. This caused the recorded patterns to be unreliable [15].

In a follow-up work [44], two different devices were fabricated and tested by the same authors: device A, which comprises of a generic Tin Oxide (SnO_2_) chemiresistive gas sensor connected to a bundle of cylindrical channels, with diameters of 50 μm and length of 5 cm; and device B, composed by a similar gas sensor connected to a single channel of much larger diameter (1000 μm) and shorter length (2 cm). The operation method is identical to the previous work reported by the authors [10], with the open ends of both channels connected to the analyte-filled chamber. Two parameters were found to be determining: D and α, related to the diffusivity in air and the interactions between the channel wall and the analyte molecules, respectively. An optimal detection method was developed by combining the two devices. Device B was employed for the determination of the analyte’s D (since D is more prevalent than α in device B due to the lower surface area that arises from the presence of a unique channel). Device A was utilized for the determination of the analyte’s α. This method allowed for the creation of a two-dimensional α-D feature space where all the tested analytes were classified. Hence, the addition of a second channel with a larger diameter (1 mm) was shown to facilitate and improve the identification process, by assigning each analyte to a specific D and α, placing them in a feature space with physiochemically meaningful axes. The two-channel types system permitted to decrease the lower VOC detection limit from 900 ppm to 500 ppm for a set of alcohols (Table 1) while for ethanol, the lowest detection level of ethanol was experimentally estimated to be 10 ppm. 

**Figure 2:**
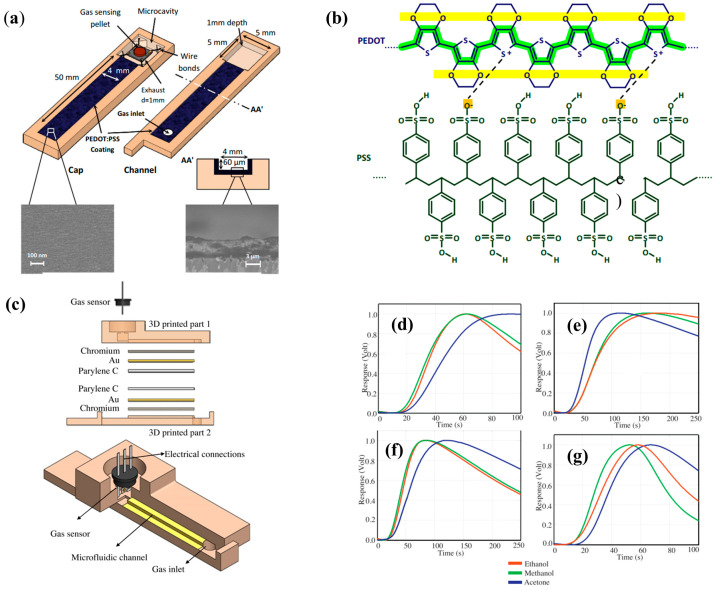
Device architecture, structure of PEDOT:PSS, normalized responses. (**a**) Scheme of the PEDOT:PSS-coated microfluidic channel integrated with a gas sensor. (**b**) Macromolecular chains constituting a PEDOT:PSS layer. (**c**) Schematic of the 3D-printed gas sensor. The channel is coated with chromium (Cr), gold (Au), and Parylene C, which was proved to be the best combination regarding selectivity. (**d–g**) Normalized responses for three different analytes for four different channel coatings. (**d**) SiO_2_ and Parylene C, (**e**) Cr-Au, (F) Cu and Parylene C, (**g**) Cr-Au and Parylene C. (**a**), (**b**) Reproduced with permission under the terms of the CC BY 4.0 license from [16] Copyright 2017, The Authors, published by Nature. (**c**), (**d–g**) Reproduced with permission from [17] Copyright 2017, Elsevier.

In order to improve the selectivity of this system, several approaches were tested in following works. Most gas sensors, including MOS, lack selectivity to different VOCs. A common approach to improve the selectivity of the device consists on chemically modifying the microchannel (µGC column) that leads the sample to the gas sensor. Hossein-Babaei et al. [16] reported the fabrication of a microchannel with internal walls coated with the conductive polymer Poly(3, 4-ethylenedioxythiophene)-poly(styrenesulfonate) (PEDOT:PSS), presented in Figure 2a. Two devices were assembled: the control device was fabricated with an uncoated channel, while the channel of the second device was coated with PEDOT:PSS. The temporal responses of 10 different analytes were measured using both the coated and the uncoated channel. The results revealed that coating the channel walls had little to no effect on the diffusion of H_2_, CO, hexane, and benzene, as the device with coated channel walls showed similar behavior to that of the control device. However, the coating greatly reduced the diffusion of alcohols and ketones (methanol, ethanol, acetone, isopropanol, isobutanol, and 2-pentanone). This selective filtering of the channel coating can be explained by the hydrogen bonds that were formed between the analytes and the coated walls: alcohols and ketones formed hydrogen bonds with the walls and thus were filtered, while the other analytes were not. Therefore, microchannels with PEDOT:PSS coated walls can be utilized as an effective alcohol and ketone filter to tune selectivity.

Continuing on this trend, Paknahad et al. [17] developed a similar type of device where the performance of 11 different channel coating combinations was tested. To understand the effect of channel dimensions on the selectivity and recovery time, different channel geometries were also investigated. In this approach, the microfluidic channels were fabricated using a 3D printer and different coating combinations were tested, comprised of materials such as gold (with chromium under for adhesion purposes), copper, Cytonix (PFCM 1104V), SiO_2_, and Parylene C (poly(*p*-xylylene) polymer). It is important to have in mind that the physisorption processes are dependent not only on analyte type but also on the material of the channel surface. The optimal coating combination was found to be that of Cr-Au and Parylene C (Figure 2c) since it offered the best results both in terms of selectivity (between ethanol, methanol, and acetone), displayed in Figure 2d–g, and recovery time when compared to other coating options. With the optimal coating determined, six target gases were analyzed (2-pentanol, methanol, ethanol, acetone, 2-butanone, and 2-pentanone) at eight different concentrations each, ranging from 250 to 4000 ppm.

The previously mentioned adsorption and desorption phenomena, and hence the selectivity of the sensor, can be tuned not only by the coating of the microchannel but also by the channel’s surface roughness. One possible approach to change the topography of the walls, is the integration of molecularly imprinted polymer nanoparticles (MIP NPs) into the channel, as reported by Janfaza et al. [45]. MIPs are synthetic polymers that possess selective molecular recognition properties. Their size, shape, and functionalities can be tailored according to the target molecules. In this case, the MIP NPs were synthesized with acetone recognition sites and were integrated into a 3D-printed dual-channel platform. One channel was coated with Parylene-C and the MIP NPs, and the other was coated only with Parylene-C (control channel), with the goal of creating a highly selective device for the recognition of acetone. The obtained results showed that the presence of MIP NPs improves the selectivity (versus the control channel), as the analytes present in the samples of acetone, butanone, and ethanol (similar size and shape among the three) revealed higher affinity to the MIP NPs, leading to higher adsorption rates and lower diffusion rates along the channel (when compared to methanol, acetonitrile, and toluene). This ultimately led to distinct temporal responses between the analytes. Hence, the implementation of MIP-coated microchannels can be used to add another degree of selectivity to the device.

### 2.2. Other Approaches Using MOS Sensors

This section addresses systems found throughout the literature that also use MOS sensors for artificial olfaction, but do not use µGC columns. The different approaches are listed in Table 2, and various parameters are presented, such as the system architecture, sensing material, range of detection and LOD, and the state of development of the sensor.

**Figure 3:**
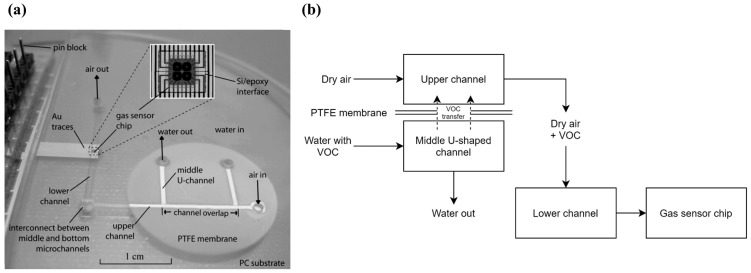
Photograph and schematic of the system. (**a**) Photograph of the fabricated device. Reproduced with permission from [19] Copyright 2007, Elsevier. (**b**) Simplified schematic of the system.

Zhu et al. [19] developed a two-phase microfluidic monitoring system to detect VOCs diluted in water. The system comprises a silicon-based micro-hotplate gas sensor chip as detection element, integrated into a microfluidic substrate. The microfluidic system was fabricated by hot embossing, where a bulk-etched silicon template was used to form microchannels on the surfaces of two polycarbonate (PC) substrates. Silicon-in-plastic fabrication was used to seamlessly integrate sensor chips into the polymer microfluidic substrate. This sensor chip contains four micro-hotplates, and each hotplate consists of a polysilicon resistor (heating), a SnO_2_ sensing film, and metal contacts in order to measure the conductivity of the sensing film. A photograph of the system is presented in Figure 3a. The working principle of the device is based on the sampling of a water source, transportation of VOC molecules from the aqueous into a vapor phase and carrying of the vapor phase to the gas sensor for analysis. Three layers of PC were fabricated, each containing one microchannel, resulting in an upper channel, a middle channel, and a lower channel. The water sample is injected in the inlet of the middle channel and a sample of dry air is injected in the inlet of the upper channel. The middle and upper channels meet at the channel overlapping region, where a PTFE membrane separates both channels, preventing the aqueous solution from moving into the upper channel, but allowing the VOCs present in the water sample to be transferred. Finally, dry air carries the VOCs to the bottom channel, through an interconnection, leading them to the gas sensor chip. The main focus of this study was to evaluate the sensitivity of the system to three different VOCs diluted in water, with results indicating approximate detection limits of 1 ppm for methanol, 10 ppm for toluene, and 100 ppm for 1,2-dichloroethane A simplified schematic of this setup is presented in Figure 3b.

**Figure 4:**
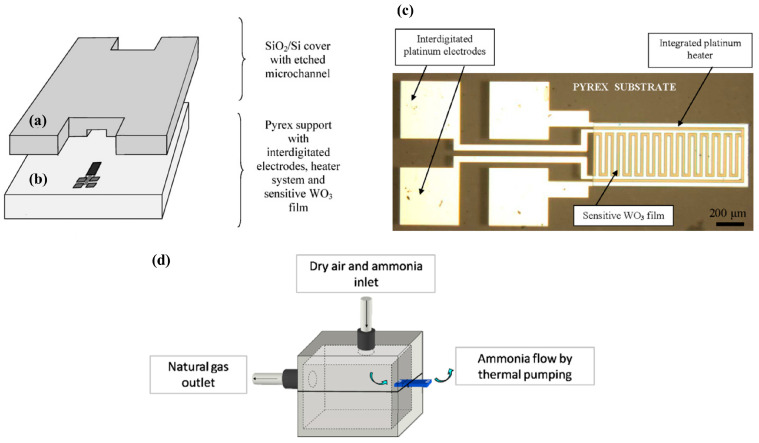
Microsensor and device assembly. (**a**) Silicon wafer with etched microchannel, and (**b**) Pyrex wafer with the integrated sensor. (**c**) The microsensor with interdigitated platinum electrodes, gas sensing film and platinum heater. (**d**) Detection chamber. Reproduced with permission from [46,47]. Copyright 2012, Elsevier.

Martini et al. [46,47] developed a compact microfluidic system with an integrated pumping system for the detection of ammonia using WO_3_ sensing films. Gas detection microfluidic devices usually rely on an external pumping system to transport the gas sample. However, this approach takes advantage of the thermal creep phenomenon to move the sample along a single microchannel. The thermal creep phenomenon can be described as the motion of gas due to a thermal gradient. The developed device is comprised of a Pyrex substrate layer and a silicon or Pyrex cover. A single 1 cm-long microchannel was etched on the cover through ultrasound etching, and a gas sensor is located at the end of the microchannel, integrated with the Pyrex substrate (Figure 4a,b). The sensor consists of a metal-oxide sensitive WO_3_ film, an integrated platinum heater, and four interdigitated platinum electrodes, as seen in Figure 4c. The heater generates the temperature gradient needed to transport the sample through the microchannel and is also used to optimize the sensor operation, as it is a metal oxide-based sensor, which requires a specific temperature to operate. After running different computer simulations, the Pyrex cover was found to be preferable (versus using a silicon cover) as it allowed an increase in thermal gradient. The microsensor response to ammonia was first tested without the Pyrex cover, using concentrations in dry air ranging from 10 to 100 ppm, which were successfully detected. The sensor resistance decreased in the presence of ammonia, which is due to the interaction of ammonia (a reducing gas) with WO3 (n-type semiconductor). Then, the thermal pumping effect was demonstrated for an ammonia concentration of 50 ppm, using a tailor-made setup that ensured that gas was only present at the channel input and flowed by thermal creep to the channel output, where the microsensor is located. In such setup, the device was placed between the interior and the exterior of a chamber (Figure 4d), with the sensor facing the exterior and the channel input facing the interior, that contained the gas sample was. A working temperature of 473 K was chosen since it provided a good balance between signal response, recovery time, and power consumption. In the future, the device should be tested for a range of ammonia concentrations in order to assess its full potential.

**Figure 5:**
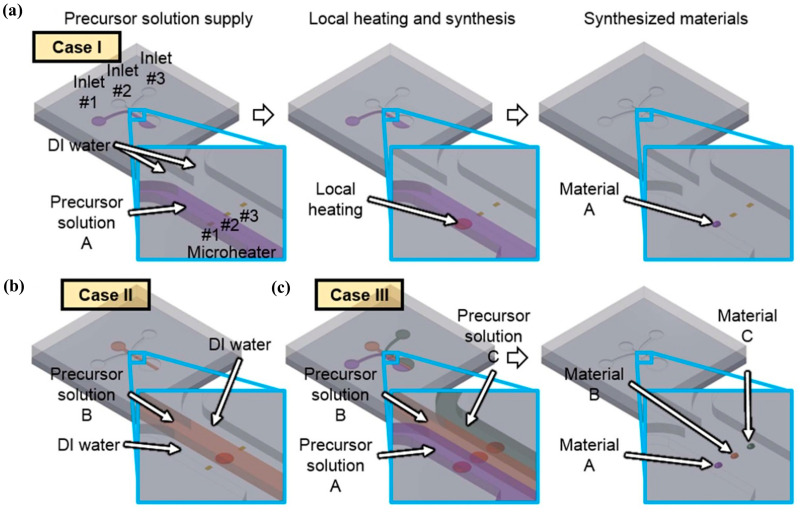
Local synthesis of nanomaterials in microfluidic channel. (**a**) selective and localized synthesis of material A on microheater 1. (**b**) Synthesis of nanomaterial B on microheater 2. (**c**) Parallel synthesis of heterogeneous nanomaterial array. Reproduced with permission under the terms of the CC-BY-NC-ND 4.0 license from [48] Copyright 2015, The Authors, published by Nature.

1D (one-dimensional) sensing nanomaterials, such as nanowires, nanorods, and nanotubes provide high surface-to-volume ratio, high sensitivity, and rapid response to analyte molecules. Research works about the application of these materials in gas sensing have increased throughout the years. For example, an approach using 1D nanomaterials was developed by Yang et al. [48] for a multiplexed gas sensor, where microfluidics was used for in situ synthesis of the sensing materials and for sensing itself. The authors developed a system comprised of three parallel microfluidic channels (fabricated through photolithography techniques) where the localized hydrothermal synthesis of nanomaterials in a liquid precursor is performed. The synthesized nanomaterials are afterwards used, in the same chip, to sense NO_2_ and CO. Micro-heaters present in the device allow for localized heat generation on each channel. Hence, when a precursor solution is heated above a given temperature, nanomaterials are synthesized (Figure 5). For example, if a certain nanomaterial is supplied to inlet 1 and deionized water is supplied to the other two inlets, while an electric bias is applied across the microheater 1, hydrothermal synthesis of the nanomaterial occurs only on the area of the microheater 1. In summary, both the synthesized material and the synthesis location can be controlled by selecting the precursor solution and the microheater, respectively. Moreover, it is possible to synthesize multiple nanomaterials at the same time, due to the independence between the channels. This is represented in Figure 5. Due to the possibility of individually supplying of multiple precursor chemicals to each channel, different combinations of sensing materials can be used. Taking advantage of this, the authors used two different combinations: a parallel array of titanium dioxide (TiO_2_) nanotubes, copper oxide (CuO) nano spikes, and ZnO nanowires (array type I) and a parallel array of ZnO nanowire/CuO nanospike hybrid nanostructures, CuO nano spikes, and ZnO nanowires (array type II). Compared to single material-based gas sensors, whose responses can be affected by interfering gases, these multiplexed sensor array devices can have enhanced selectivity and accuracy by accessing data collected from different sensing materials simultaneously. The sensing materials were exposed to 0.1–20 ppm of NO_2_ gas and 20–1000 ppm of CO gas in dry air. Since NO_2_ is an oxidizing gas and CO is a reducing gas, their interaction with the metal oxide nanomaterials results in distinct electric resistance changes. First, the sensing materials were heated (through Joule heating of the microheaters), reaching temperatures of 300–350 °C. Both arrays were tested for different concentrations of NO_2_ and CO (0.1, 0.5, 1, and 2 ppm for NO_2_; 20, 200, 500, and 1000 ppm for CO), and the relative changes of electrical resistance were measured. This approach demonstrated the possibility of using combinations of n-type and p-type semiconductor nanomaterials for a multiplexed gas sensor to tune the selectivity of the device.

### 2.3. Approaches not Using MOS Sensors

Although it is less common, microfluidic gas sensing using electrical transduction can also be achieved without resorting to MOS sensors. This section highlights two approaches that utilize different sensing methods other than MOS sensors.

**Figure 6:**
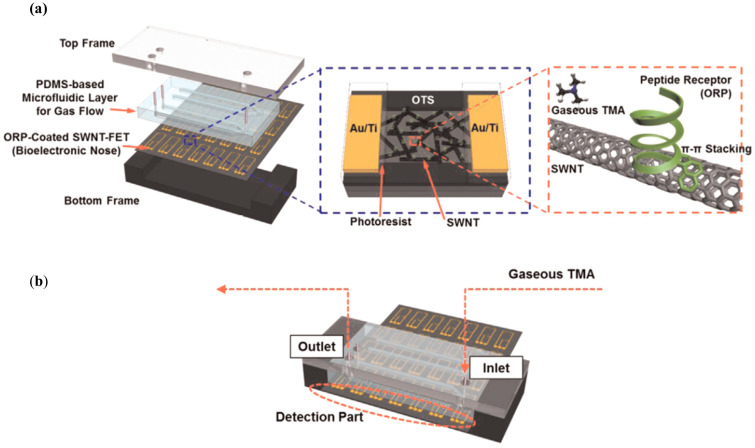
Schematic of the different parts that make up the device. (**a**) The surface of the SWNTs was functionalized with ORPs through π- π stacking. (**b**) The assembled to device for TMA sensing. To facilitate the electrical measurements, the electrodes were exposed to the outside of the platform, creating a detection part. Reproduced with permission from [38] Copyright 2015, Elsevier.

Lee et al. [38] developed a microfluidic system to monitor gaseous trimethylamine (TMA) in real-time. Trimethylamine is released from seafood during spoilage, and as the food continues to decay and deteriorate, TMA concentrations levels rise. Hence, this device was developed to serve as a detector for spoiled seafood by monitoring TMA (gaseous) levels in food samples. As seen in Figure 6a, the device is composed by four different layers from top to bottom: a top frame, a PDMS microfluidic layer for gas flow, a layer containing single-walled carbon nanotube field-effect transistors (SWNT-FETs), which are functionalized with olfactory receptor-derived peptides (ORPs), and a bottom frame. The selectivity of peptides towards target VOCs is relatively easy to tune do their small size, making them a simple biological and low-cost alternative towards selective biosensors [13]. The employed ORPs can recognize TMA molecules, and to obtain a response, the changes in the conductance between the SWNT-FET electrodes are measured in the detection part of the device. This feature can be seen in Figure 6b. In a preliminary experiment, various samples with different concentrations of TMA were tested in order to compare the response between bare and ORP-coated SWNT FETs. The conductance with bare SWNT-FETs did not change with increasing TMA concentrations, while the conductance with ORP-coated SWNT-FETs did, revealing correct immobilization of the ORPs. The response of the sensor to TMA was also compared to that of other gaseous odorants with similar chemical structures, such as triethylamine, dimethylamine, acetic acid, and acetone. The results showed much stronger responses to TMA than those generated by the other gases, which indicated the high selectivity brought up by the ORP. As for the main experiment, different foods such as oysters, mushrooms, paprika, and broccoli were ground using a food blender and stored in bottles for 3 days, after which the gas samples generated from the spoiled food were utilized. When comparing the responses between the gas samples of spoiled oysters and other spoiled foods, the gas sample from the spoiled oysters showed a significant change in conductance. These tests showcased the selectivity and efficiency of the device when assessing the freshness of seafood, with a TMA limit of detection of 10 ppt (much lower than that of other conventional TMA sensors). This excellent limit of detection compensates for the complex and intricate structure of the device. Moreover, the possibility to bind different ORPs to the SWNT-FETs makes it a highly tailorable system.

**Figure 7:**
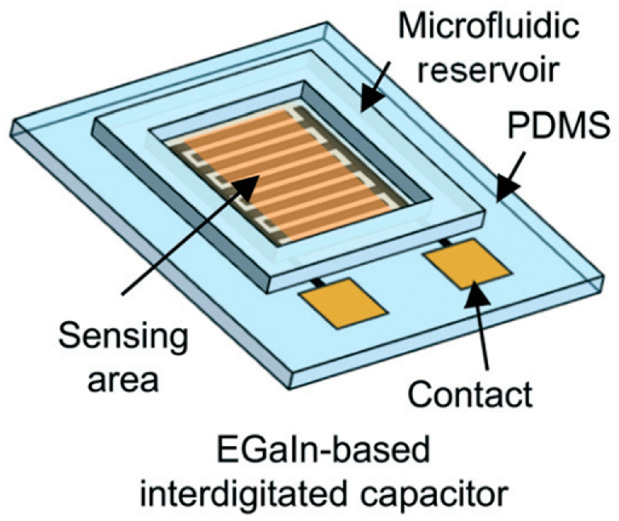
All-soft microfluidic chemical sensing platform using gallium-based liquid metal (EGaIn) and PDMS for liquid-phase and gas phase VOC detection. Reproduced with permission from [49] Copyright 2017, Royal Society of Chemistry.

In microfluidics and VOC/gas sensing, PDMS is generally used as a substrate in the channel platform to transfer the sample from its source to the sensing area, through microchannels. An approach by Kim et al. [49] takes advantage of PDMS not only as a substrate material but also as a sensing material. The authors developed an all-soft microfluidic sensing platform based on EGaIn (eutectic gallium-indium alloy), which is a liquid metal, and PDMS for sensing of both liquid- and gas-phase VOCs. The fabricated device is comprised of a PDMS substrate with an EGaIn-based interdigitated capacitor and a PDMS microfluidic reservoir. The device, presented in Figure 7, was fabricated via an advanced liquid metal patterning technique based on soft lithography. With the main goal of this approach being the development of an all-soft sensor, electrical characterization was performed on the device while applying bending and twisting forces. The capacitance was shown to remain stable under both deformation types. Relative permittivity (ε_r_), diffusion coefficient (D), and partition coefficient (K) for the VOCs in the PDMS sensing film all contribute to the measured capacitance signal. Different concentrations of three distinct VOCs were tested – ethanol, methanol, and isopropanol – and their effect on the relative capacitance change of the device was measured. The results showed, for all VOCs, that higher concentrations led to increased capacitance changes. Positive capacitance changes can be attributed to the higher relative permittivity of the analytes when compared to that of PDMS. Selectivity between methanol and the other VOCs was proven to be very high, however, the signal response to isopropanol and ethanol was very similar, revealing poor selectivity between the two high, however, the signal response to isopropanol and ethanol was very similar, revealing poor selectivity between the two.

## 3. Microfluidic Gas Sensing Devices Using Optical Transduction

Methods that use electrical transduction, which were mentioned above, usually lack the ability to recognize individual compounds, and as such, quantitative measurement of each gas sample is difficult to achieve [18,27]. In this section, we present representative examples of microfluidics systems with optical transduction of the gas sensing events. Several optical transduction methods are covered, namely UV spectroscopy, luminescence, and fluorescence. 

**Figure 8:**
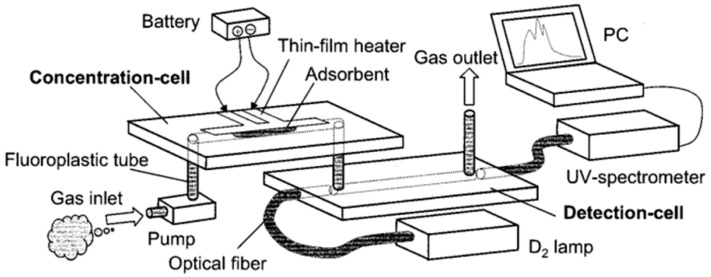
Simplified schematic representation of the device, comprised of a pump, a concentration cell (containing a thin film heater and an adsorbent), a detection cell, and the optical apparatus (UV lamp, optical fiber, and UV spectrometer). The target gas sample is first introduced into the concentration cell. Once inside the concentration cell, the gas is adsorbed on the adsorbent present in the cell. After reaching the desired sample concentration, the temperature of the thin-film heater is raised to approximately 200 °C (by voltage application) in order to desorb the concentrated gas from the adsorbent, which is transferred into the detection cell (this high temperature might be problematic when using for other sensing applications). Reproduced with permission from [18]. Copyright 2001, American Chemical Society.

Ueno et al. [18,26,27,28,29,30], designed an integrated system that includes a microfluidic device for the detection of airborne BTEX (benzene, toluene, ethylbenzene, and xylenes) with the use of UV spectroscopy. The setup of this system (illustrated in Figure 8) consists of a gas pump, a concentration cell, a detection cell, and an optical system comprised of a lamp and a UV spectrometer.

Both the concentration and the detection cells are made of Pyrex, with 3 cm × 1 cm. Photolithography techniques were used for the deposition of a platinum electrode on the concentration cell, to act as a thin-film heater. A small amount of amorphous silicon dioxide power was introduced in the microchannel of the concentration cell, to serve as adsorbent material. Silicon dioxide is suitable as an adsorbent material due to its strong interaction with BTEX gases. The detection cell contains two small holes and microchannel, where optical fiber was inserted through it with one end of the fiber connected to a 30 W deuterium (D_2_) lamp and the other connected to a UV-spectrometer. By having the lamp and the UV spectrometer aligned with the detection cell, the time-dependent UV absorption spectra of the gas sample can be measured. The analysis of the specific peaks on the spectra enables the identification of each compound present in the sample. However, the system is comprised of two separate cells, and other moving parts such as the tubing. This can lead to sample leakage and therefore loss of sensitivity. Ueno’s device was further modified by improving different aspects of its architecture, namely regarding cell structure, sample pre-processing and delivery (Table 3).

The first modifications concerned the adsorbent, the cell structure, and the gas transfer system [29,30]. Mesoporous silicate powder (SBA-16) was used as an adsorbent to concentrate the VOCs, which resulted in the successful detection of 100 ppb of benzene with a preconcentration time of 30 min [30]. Optimization of the detection cell was accomplished by coating the inside of the cell’s microchannel with a reflective layer of platinum, increasing both the waveguide efficiency and the signal-to-noise ratio (SNR) [29]. By using the newly tested SBA-16 adsorbent and the improved detection cell, 25 ppb benzene gas detection was achieved with a preconcentration time of 50 min. The gas transfer system of the device was also improved [29]. In the previous designs, there was only one pumping system, which was used to sample the air and transfer the gas from the concentration cell to the detection cell. The integration of an additional pump and a solenoid pinch valve resulted in further improvement of the SNR.

**Figure 9:**
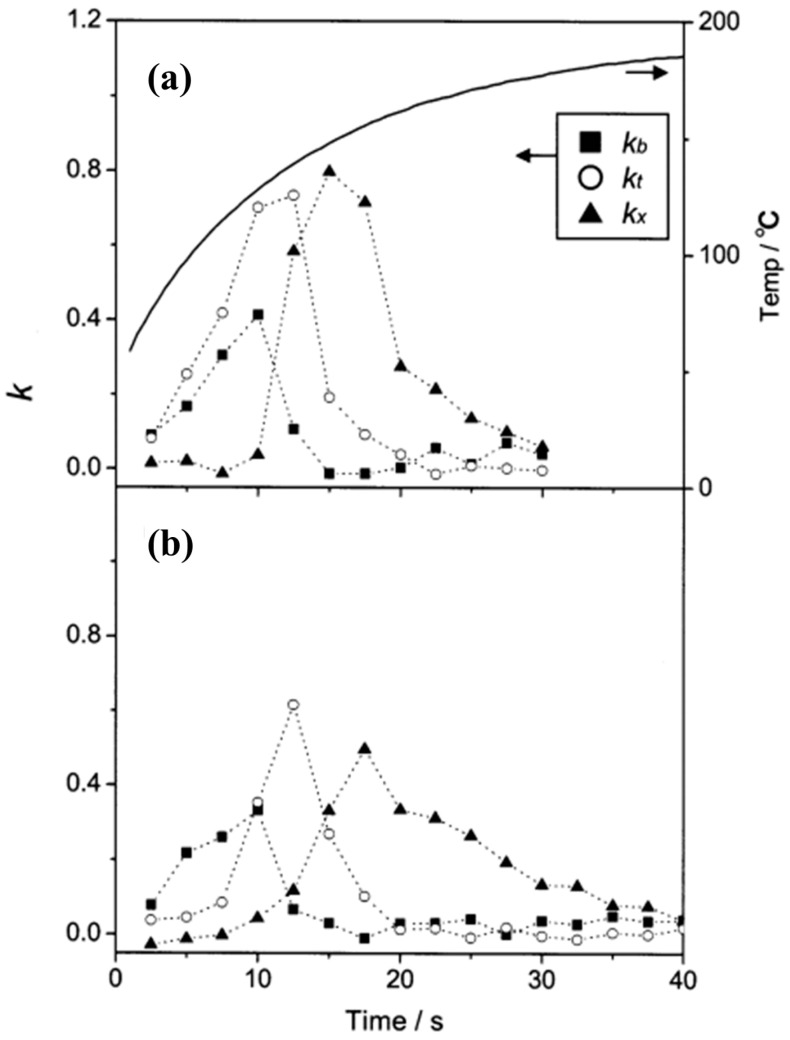
Changes in kb, kt, and kx (coefficients that correspond to the ratio of benzene, toluene, and *o*-xylene in the mixture gas, respectively) against time when SDP (**a**) and SBA-15 (**b**) are used. The thermal characteristic of the concentration cell with SDP is superimposed on the plot (boldface line). The thermal characteristic of the concentration cell with SBA-15 was not presented as it was very similar to that of SDP. Reproduced with permission from [27]. Copyright 2002, American Chemical Society.

Detection of the individual components of a BTEX mixture was also tested by Ueno et al. [27,28]. A BTEX mixture gas was prepared by diluting commercial-grade toluene (50 ppm), benzene (50 ppm), and o-xylene (50 ppm) gases with nitrogen. The detection procedure was similar to that of the previous approach, but this time two different adsorbents were tested: amorphous silicon dioxide powder (SDP) and mesoporous silicate powder (SBA-15). The changes in the absorption spectra when using either SDP or SBA-15 as absorbents were obtained. SBA-15 proved to be the best adsorbent as it provided better separation for each component of the BTEX mixture (Figure 9). This was attributed to the differences in the thermal desorption properties of each adsorbent, caused by their different pore structures.

A couple of experiments were performed in order to improve the sensitivity of this method. To assess the advantage of having a concentration cell, the limit of detection of the device with and without this cell was determined. The results revealed that with the concentration cell, the limit of detection was found to be 4 ppm (for toluene). On the other hand, the limit of detection without the concentration cell—by supplying the original gas directly to the detection cell during the spectral measurement—was determined to be 100 ppm. Hence, the integration of a concentration cell prior to the detection cell was proven to be very advantageous as it enabled the detection of a toluene gas concentration about 25 times lower than when measured with the detection cell alone.

**Figure 10:**
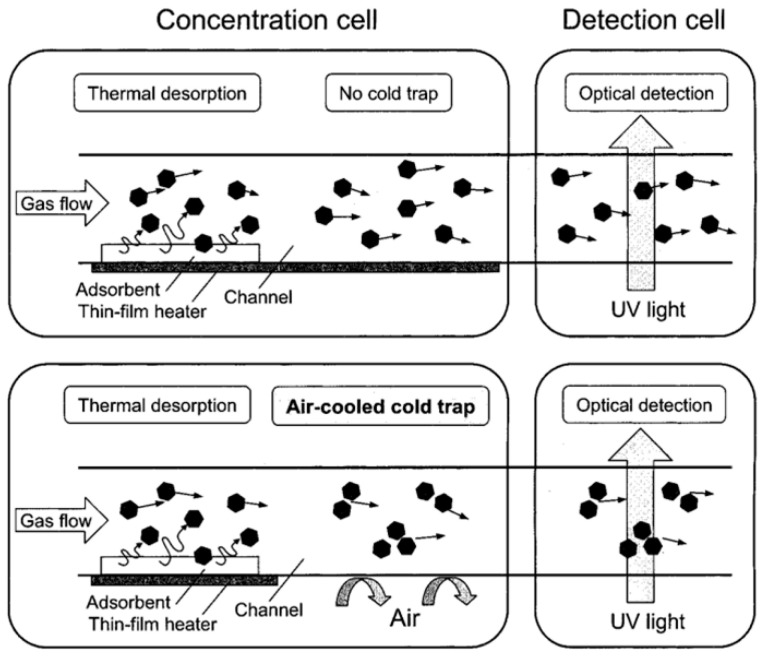
Schematic illustrating the effect of the integration of the CT in the concentration cell. Reproduced with permission. [26] Copyright 2002, American Chemical Society.

Another modification was the integration of a cold trap (CT) [26,28] in the concentration cell, downstream of the adsorbent. This helps to maintain high gas concentrations in the concentration cell just before introducing the gas into the detection cell. This mechanism is presented in Figure 10. By implementing a cold trap, the limit of detection for toluene was improved to 0.05 ppm. To obtain a faster response (thus a sharper pulse), the Teflon connection tube that separated the concentration and detection cell was removed, and the two cells were joined [28]. This approach resulted not only in a faster response (2.5 s faster than that of the separated device) but also in a lower limit of detection for toluene (0.01 ppm). However, it also led to wider response curves, which was attributed to the conflict between the integration and the cold trap. The authors predict that this can be resolved by forming a space between the two cells or by integrating both cells in one single chip.

**Figure 11:**
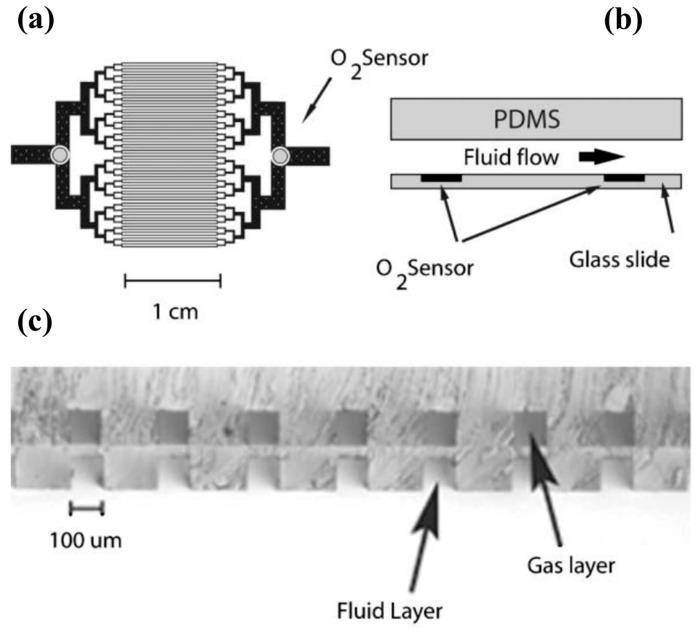
Schematic of the device and photo of the two layers. (**a**) Lithography mask of the capillary structure, where the circles represent the location of the O_2_ sensors. (**b**) Cross section of the microfluidic device. (**c**) Vertical alignment between gas and fluid channels is noticeable in the cross-section view. The channels are separated by a 20 μm membrane. By having a thin PDMS membrane between the two layers, diffusion between the two phases is possible (due to the permeability of PDMS to oxygen). Reproduced with permission from [36] Copyright 2005, Royal Society of Chemistry.

Luminescent chemical sensors have generated interest due to their fast, linear response. A luminescent oxygen sensor incorporated into a PDMS microfluidic oxygenation platform was developed by Vollmer et al. [36] for the detection of gaseous or dissolved oxygen and can be seen in Figure 11. The principle in place is the excitation of a luminescent dye integrated with a polymer matrix. By having a LED above the sensor and a photodiode below it, oxygen detection is possible due to the high degree of transparency of the PDMS. The chemical dye used by the authors was Pt octaethyl-porphyrin-ketone (PtOEPK), which is suitable for oxygen detection. PtOEPK exhibits an absorption peak at 590 nm in the visible spectrum, and its emission peak is at 760 nm, which is detectable by Si photodiodes. By using a mass transfer model, which the authors named “parallel channel geometry”, the mass transfer properties of the microfluidic oxygenator were characterized. In the device, oxygen diffusion occurs from the O_2_ gas reservoir into the fluidic channel, through the PDMS membrane. The limit of detection of the device was determined to be 120 ppb.

**Figure 12:**
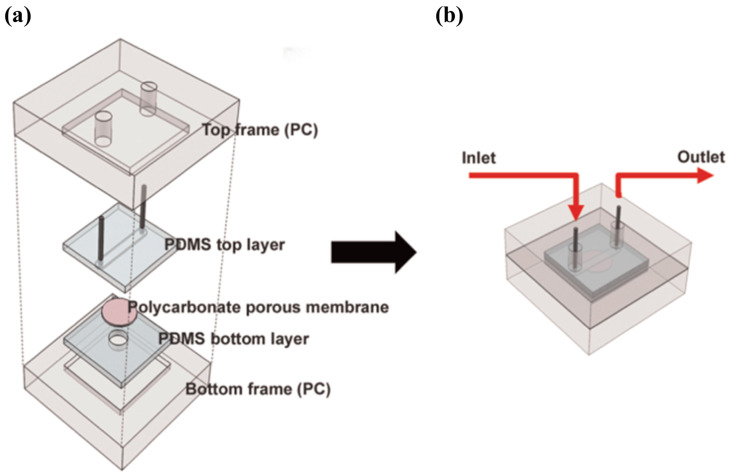
Schematic of the microfluidic system. (**a**) Device assembly. (**b**) Assembled microfluidic device and injection process. Reproduced with permission from [50] Copyright 2015, Elsevier.

An approach based on the change of fluorescence signals was developed by Lee et al. [50]. The authors created a microfluidic device for the detection of gaseous odorants where cells expressing human olfactory receptors (hORs) were cultured in a polycarbonate porous membrane. The device is comprised of two PDMS layers, which are sealed between two polycarbonate (PC) frames, and a porous membrane (2 μm-diameter pores), which sits between the PDMS layers. This is illustrated in Figure 12. Human embryonic kidney-293 (HEK-293) cells expressing different hORs were cultured on a polycarbonate membrane and were then loaded with Fluo-4 AM, a calcium indicator. After assembly, gaseous odorants such as bourgeonal, β-citronellol and geraniol (2 ppm) were injected into a microfluidic channel created on the top PDMS layer. The cellular response was monitored through the change of fluorescence signals with Fluo-4 AM, using a fluorescence microscope. Different hORs recognized distinct gaseous odorants, resulting in a high degree of selectivity. This approach showed great potential and, according to the authors, can in the future be utilized in areas such as medical diagnosis, gas sensing, and even detection and production of fragrances and flavors.

## 4. Other Approaches

**Figure 13:**
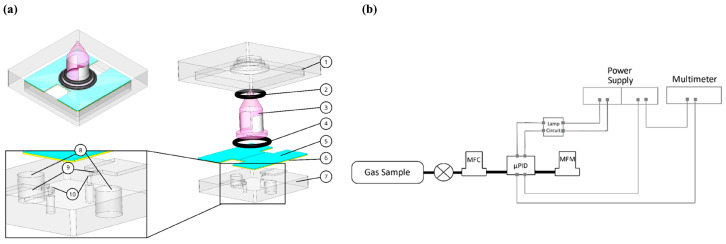
Device architecture and schematic of the different components. (**a**) Assembled and exploded view of the detector core. (1) Top poly(methyl methacrylate) (PMMA), (2) O-ring 1, (3) UV lamp, (4) O-ring 2, (5) shield, (6) copper electrodes, (7) bottom PMMA, (8) electrode connection holes, (9) elevation structure, (10) micro-pores. (**b**) Experimental setup used for signal measurements from µPID prototype. Reproduced with permission under the terms of the CC BY 4.0 license from [12] Copyright 2019, the authors, published by MDPI.

This section addresses VOC sensing devices that do not fit the categories described so far. VOC detection through photoionization has been reported by Rezende et al. [12] for sensing toluene, with a limit of detection of 0.6 ppm. The working principle of a photoionization detector (PID) is based on the ionization of gaseous compounds by a light source: a gas sample enters the ionization chamber, where the molecules of the sample are ionized by the photons emitted by the ionization source. Miniaturization of the ionization chamber is of major importance since it should not only improve the sensitivity of the signal but also decrease the amount of sample needed, which results in faster analysis time. In this approach, displayed in Figure 13, the ionization chamber is the microchannel formed by the two copper electrodes, the top and bottom PMMA plates, and the UV lamp. 

The volume of this μPID can be up to 100 times lower than the one of commercial PIDs. The height of the microchannel is defined by the height of the copper electrodes. As for the shape of the electrodes, three designs were tested in order to find the optimal ionization chamber volume. The coating of the copper electrodes is also a relevant matter since the high-energy radiation of the UV lamp can damage the electrodes and cause signal noise. Two materials were considered for the coating, such as diamond-like carbon (DLC) and PMMA. The latter was found to be more stable. To compare the three channel designs, the detection of nitrogen and toluene at 100 ppm was analyzed. It was found that the μPID signal increases with the flow rate and the electrode area. The design with highest chamber volume and electrode area offered the best sensitivity and selectivity between nitrogen and toluene. Hence, more experiments were performed with this design, using three different toluene concentrations, ranging from 1 to 100 ppm. The results revealed a linear response, with higher concentrations of toluene resulting in increased current signals. Due to the complexity of this device, with all of its moving parts, practicality might be an issue when utilizing it.

**Figure 14:**
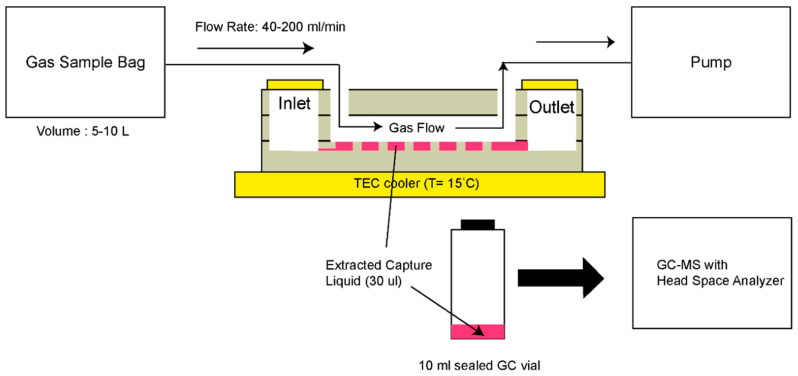
Schematic representing the gas sampling method. Reproduced with permission under the terms of the CC BY 4.0 license from [37] Copyright 2019, the authors, published by MDPI.

Although GC-MS was utilized for detection, an interesting approach using microfluidics was developed by Warden et al. [37]. Gas-to-liquid extraction was promoted through the design of an open capillary channel incorporated into a microfluidic platform. The device, fabricated via typical photolithography techniques, is composed of three layers of borosilicate glass bonded to each other, with an inlet and outlet for the liquid phase, and two ports for the gas sample. A schematic of the device is presented in Figure 14. As for the operation procedure, a capture liquid is injected in the inlet and eventually reaches the open channel network through capillary action. The open channel network consists of arrays of hexagonal protrusions with space between them for the liquid to settle. The target gas sample is inserted in one of the gas ports and when it reaches the central area of the device (open channel network), the VOCs in the gas phase are transferred to the capture liquid. Finally, the capture liquid is collected for analysis via the outlet with a syringe. The tested VOCs were hexanal and allyl methyl sulfide, which are known as breath biomarkers for lung cancer and malaria, respectively. Alpha-cyclodextrin in Milli-Q water was used as the capture liquid. Like mentioned before, GC-MS was used for VOC detection. However, the authors predict that detection can be done using solely a microfluidic system.

## 5. Conclusions and Future Perspectives

VOCs are compounds with high vapor pressure and low water solubility, which can originate from several different sources. The gold standard for VOC detection lies in the pairing of gas chromatography and mass spectrometry (GC-MS). However, this technique possesses many disadvantages, and microfluidics-based devices allow to circumvent them, as it offers the ability to utilize small quantities of reagents and samples, high resolution and sensitivity of analysis, low fabrication and operation costs, short analysis time, and the ability to perform in situ operation. Present in this review is a vast array of approaches regarding VOC sensing using microfluidics. The two main methods of signal transduction were covered – electrical and optical – and details for each approach were provided, regarding the structure of the devices, the tested VOCs, the limits of detection of the devices, and the different sensing materials that were used. 

Throughout the literature, the great majority of the approaches utilize electrical transduction, and the coupling of µGC columns and MOS gas sensors is one of the most popular device architectures. Measurement simplicity, durability, and low fabrication costs makes MOS sensors an appealing solution for microfluidic artificial olfaction, despite having high energy consumption resulting from elevated operating temperatures [39]. Generally, MOS sensors have a subpar ability to recognize individual compounds, and as such, quantitative measurement of the VOCs present in complex mixtures is difficult to achieve [18,27]. In this case, methods that use optical transduction might perform better. Devices that use optical detection methods provide high sensitivity and stability to environmental factors, but also have drawbacks – the majority requires optical fiber, which makes them difficult to miniaturize [39]. In this category, the analyte detection is measured through UV light absorption spectra [18,27,28,29,30]. 

In the literature, the discrepancy between methods using electrical and optical transduction is steep, and more optical methods will hopefully be developed in the near future. LOD as low as 10 ppt were achieved [38], although the majority of the approaches provided LOD in the ppm and ppb range. The methods present in the literature provide many options regarding sensing materials, such as SnO_2_ [14,15,16,17,19,44], WO_3_ [46,47], chemical dyes [36], ZnO/CuO hybrid nanostructures, CuO nanospikes, ZnO nanowires [48], ORP-coated SWNTs [38], and PDMS [49]. Several methods were only applied to a specific VOC/gas, such as oxygen [36], toluene [12], TMA [38], and ammonia [46,47], and will hopefully soon be adapted to a wider range of compounds. Improvements to existing devices were also addressed, which have the goal of increasing the selectivity or sensitivity of the devices. For example, several works mentioned the coating of microchannels and the use of µGC columns in order to improve selectivity to specific target gases [16,17,45]. Regarding the concentration/detection cell system, discussed in the optical transduction section, different adsorbent materials were tested, and the selectivity of the device was improved. This resulted from the changes in the absorption spectra when using the new adsorbents, ultimately providing better separation between each compound of the sample mixture. To improve the sensitivity of the system, different approaches were also tested, such as the implementation of a cold trap in the concentration cell [26,28], and the integration of the two cells, eliminating the tubing between them [28]. This resulted in increased gas concentrations (preventing gas dilution) and sharper pulses in the absorption spectra, respectively. Of course, when it comes to sensing devices, practicality is a major concern. Several systems present in this review can provide it, by having few moving parts [14,15,16,17,44,45,46,47], while others have more complex structures [12,19,38]. Intricate systems can ultimately pay-off, either by allowing for the sensing of different samples or by providing excellent LOD [38]. 

The field of artificial olfaction and gas-sensing is a very promising one [51,52], and throughout the literature, there is a strong indication that the integration of microfluidic technology with these devices is very advantageous. As seen in this review, the future of the field seems to be heading towards the integration of miniaturized GC components and MOS sensors, since the use of µGC circumvents many issues that exist in conventional GC, and the advantages of MOS technology normally outweigh its drawbacks. As for the future of microfluidics, a lot of works still needs to be done. For one, the field of microfluidics is still largely based on proof-of-concept work and academic/research papers, and it must become successful commercially in order to thrive. Also, more bridges need to be built between the engineers and physicians who develop the devices and the end-user (such as biologists, public health officials, and clinicians). By achieving this, new ideas and approaches will surely be created as the communication and collaboration between the two parties evolves [34,53]. Regardless, from the cutback on overall costs to the reduction of sample quantity, the use of microfluidics in this field shows great potential to further advance the area of artificial olfaction.

## Figures and Tables

**Table 1:** Devices coupling a µGC column and commercially available MOS sensors.

μGC Column Coating	Sensing Material	Analytes	Range of Detection; LOD	Refs
No coating	Tin oxide (SnO_2_)	Hydrogen, carbon monoxide, argon, oxygen, methanol, ethanol, isopropanol, 1-propanol, *tert*-butanol, 2-butanol, *iso*-butanol, 1-butanol, methane, *n*-butane, *n*-pentane, acetone, butanone, 2-pentanone, methyl isobutyl ketone, chloroform, toluene, benzene, carbon tetrachloride, and ammonia	900–1100 ppm; -	[15]
No coating	Tin oxide (SnO_2_)	Ethanol	500–1000 ppm; 10 ppm	[44]
		Methanol, *iso*-propanol, 1-propanol, *tert*-butanol, *iso*-butanol, 2-butanol, 1-butanol.	500–1000 ppm; -	
PEDOT:PSS	Tin oxide (SnO_2_)	H_2_, CO, methanol, ethanol, 2-propanol, *iso*-butanol, acetone, 2-pentanone, hexane, benzene	1000–10,000 ppm; -	[16]
No coating	Tin oxide (SnO_2_)	Acetone, hydrogen, ethanol, and benzene	250–3000 ppm; -	[14]
Combinations of Cr, Au, Cu, and Parylene C	Tin oxide (SnO_2_)	Methanol, ethanol, 2-pentanol, acetone, 2-butanone, 2-pentanone.	250–4000 ppm; -	[17]
MIP NPs	Tin oxide (SnO_2_)	Acetone, ethanol, methanol, butanone, acetonitrile, toluene	200–4000 ppm; -	[45]

LOD—Limit of detection; PEDOT:PSS—poly(3,4-ethylenedioxythiophene) polystyrene sulfonate; MIP NPs—molecularly Imprinted nanoparticles.

**Table 2:** Other VOC sensing systems that use MOS sensors (and no µGC column).

System Architecture	Sensing Material	Analytes Tested	Range of Detection; LOD	Measurement	State of Development of the Sensor	Refs
2-phase microfluidic water monitoring system	SnO_2_ sensing film	Methanol	0–100 ppm; 1 ppm	Conductance	Research level	[19]
		Toluene	0–100 ppm; 10 ppm			
		1,2-dichloroethane	0–1000 ppm; 100 ppm			
Pyrex substrate coupled with a silicon cover (etched microchannel)	Sensitive WO_3_ film	Ammonia	10–100 ppm;	Resistance	Research level	[46,47]
Parallel supply of multiple precursor chemicals within microfluidic channels	ZnO/CuO hybrid nanostructures, CuO nanospikes, and ZnO nanowires	NO_2_	0.1–20 ppm; 0.1 ppm	Resistance	Research level	[48]
		CO	20–1000 ppm; 20 ppm			

ORP—olfactory receptor-derived peptides; SWNT-FETs—single-walled carbon nanotube field-effect transistors.

**Table 3:** Architectures developed to improve the performance of the concentration and detection cell system by Ueno et al.

System Improvements	Adsorbent Material	Analytes	LOD	LOD Improvement	Refs
-	Amorphous silicon dioxide powder (SDP)	Toluene	4 ppm	-	[18]
-	Mesoporous silicate powder (SBA-15)	Benzene	1 ppm	4-fold	[30]
	Mesoporous silicate powder (SBA-16)		100 ppb	40-fold	
Optimized gas transfer system, increase of signal-to-noise ratio	Mesoporous silicate powder (SBA-16)	Benzene	10 ppb	400-fold	[29]
Separate detection of the components of BTEX mixture gas (improvement of thermal desorption characteristics)	Mesoporous silicate powder (SBA-15)	Mixture of toluene, benzene, and *o*-xylene	1 ppm	4-fold	[27]
Integration of a cold trap (CT)	Amorphous silicon dioxide powder (SDP)	Toluene	0.05 ppm	80-fold	[26]
Integration of a cold trap (CT), joined the two cells	Mesoporous silicate powder (SBA-15)	Toluene	10 ppb	400-fold	[28]
